# Vestibular Function in CANVAS Patients: Qualitative and Quantitative vHIT Analysis

**DOI:** 10.3390/audiolres16050151

**Published:** 2026-10-01

**Authors:** Pasqualina Maria Picciotti, Rolando Rolesi, Gabriella Silvestri, Salvatore Rossi, Giorgia Rossi, Jacopo Galli

**Affiliations:** 1 Department of Head and Neck and Sensory Organs, Università Cattolica del Sacro Cuore, 00168 Rome, Italy; pasqualinamaria.picciotti@unicatt.it (P.M.P.);; 2 Otorhinolaryngology Unit, Department of Neurosciences, Sensory Organs and Thorax, Fondazione Policlinico Universitario Agostino Gemelli IRCCS, 00168 Rome, Italy; 3 Department of Neurosciences, Università Cattolica del Sacro Cuore, 00168 Rome, Italy; 4 Neurology Unit, Department of Neurosciences, Sensory Organs and Thorax, Fondazione Policlinico Universitario Agostino Gemelli IRCCS, 00168 Rome, Italy

**Keywords:** CANVAS, RFC1, video Head Impulse Test, vestibulo-ocular reflex, corrective saccades, vestibular ganglionopathy

## Abstract

**Background/Objectives:** Vestibular dysfunction is an important feature of cerebellar ataxia, neuropathy, and vestibular areflexia syndrome (CANVAS) associated with biallelic *RFC1* expansions. This study aimed to characterize both quantitative and qualitative vHIT findings in genetically confirmed CANVAS patients. **Methods:** Fifteen patients with genetically confirmed CANVAS underwent a clinical neuro-otological assessment including horizontal video Head Impulse Test (vHIT). Quantitative analysis included vestibulo-ocular reflex (VOR) gain measurements, while qualitative analysis focused on corrective saccade characteristics, including number, peak velocity, latency interval, temporal organization, and predominant compensatory pattern. Correlations between vestibular parameters, disease duration, and clinical severity assessed using the Scale for the Assessment and Rating of Ataxia (SARA) were explored. The study specifically focused on the quantitative and qualitative characteristics. **Results:** All patients exhibited bilateral vestibular dysfunction, with markedly reduced VOR gains and generally low interaural asymmetry, confirming severe and largely symmetric vestibular hypofunction. Disease duration negatively correlated with VOR gain and positively correlated with the number of corrective saccades. Lower VOR gains were significantly associated with larger corrective saccades and wider latency intervals, while saccadic amplitude and latency were positively correlated. Qualitative analysis revealed a consistent pattern of predominantly overt, temporally dispersed (anisochronous) corrective saccades, with no evidence of predominant covert responses or isochronous clustering. The relationship between vestibular impairment and clinical severity was variable, suggesting partially independent progression of vestibular and cerebellar dysfunction. **Conclusions:** In this cohort of genetically confirmed CANVAS patients, severe, bilateral, and largely symmetric vestibular hypofunction was observed, together with a predominantly overt and temporally dispersed corrective saccade pattern. Qualitative analysis of horizontal vHIT responses may provide complementary information to conventional VOR gain measurements and may contribute to a more detailed characterization of vestibular and oculomotor findings in RFC1-related disease.

## 1. Introduction

Inherited ataxias are clinically and genetically heterogeneous neurodegenerative disorders mainly involving the cerebellum and its pathways. Recently, biallelic intronic expansions in Replication Factor C subunit 1 gene (RFC1) have been shown to be associated with an autosomal recessive (AR) ataxia named RFC1 disease spectrum or CANVAS (Cerebellar Ataxia, Neuropathy, Vestibular Areflexia Syndrome, OMIM #614575) when all three prominent neurological features are present [1]. The disease exhibits a slowly progressive course, with variable onset and evolution of vestibular, cerebellar, and sensory symptoms over several decades. 

Current evidence suggests that vestibular dysfunction in CANVAS primarily reflects a sensory neuronopathy involving Scarpa’s ganglion rather than primary damage to vestibular end organs or central vestibular nuclei. Histopathological studies have demonstrated degeneration of vestibular afferents innervating the semicircular canals, resulting in severe bilateral vestibular hypofunction [2]. Consequently, vestibular assessment represents a fundamental component of both diagnosis and clinical characterization.

Vestibular dysfunction in CANVAS is typically evaluated using complementary tests that probe different frequency domains of the vestibulo-ocular reflex (VOR). The video Head Impulse Test (vHIT) assesses high-frequency VOR function and consistently demonstrates markedly reduced gains with frequent corrective saccades in most patients [3,4,5]. In contrast, caloric testing evaluates low-frequency vestibular responses and often reveals severe bilateral hypofunction or areflexia, supporting a widespread impairment of vestibular afferents [6]. Additional dynamic measures such as the visually enhanced vestibulo-ocular reflex (VVOR), which reflects the integration of vestibular input with visual and cerebellar mechanisms during low-frequency head movements, have also shown significant reduction in CANVAS [7,8]. Taken together, these findings indicate that CANVAS is characterized by a broad-spectrum vestibular deficit affecting both high- and low-frequency VOR pathways as well as visuo-vestibular integration mechanisms.

Although quantitative VOR gain abnormalities are well established, less attention has been devoted to the qualitative analysis of corrective saccades generated during vHIT. Corrective saccades represent a key compensatory mechanism for maintaining visual stability in patients with vestibular failure. Beyond gain reduction, parameters such as saccadic latency, amplitude, and temporal distribution may provide additional information regarding the severity of vestibular deafferentation and the effectiveness of central compensatory mechanisms. This aspect may be particularly relevant in CANVAS, where vestibular dysfunction coexists with cerebellar degeneration and may therefore influence the organization of compensatory eye movements.

Previous studies have primarily focused on quantitative vestibular deficits [5], whereas the qualitative characteristics of vHIT responses in CANVAS remain poorly characterized. A more detailed assessment of corrective saccadic behavior may therefore contribute to a better understanding of vestibular and oculomotor dysfunction in this disorder.

The aim of the present study was to characterize both quantitative and qualitative vHIT findings obtained from horizontal vHIT in patients with genetically confirmed CANVAS. In addition to VOR gain, we evaluated corrective saccade number, peak velocity, latency interval, temporal organization, and predominant compensatory pattern, and explored their associations with reported disease duration and clinical severity assessed using the Scale for the Assessment and Rating of Ataxia (SARA). Given the retrospective and cross-sectional nature of the study, these analyses were considered exploratory and were intended to generate hypotheses regarding the relationship between vestibular impairment and compensatory oculomotor responses in RFC1-related disease.

## 2. Materials and Methods

**Study Design and Participants**: We conducted a retrospective, single-center observational study on patients diagnosed with CANVAS in neurological follow-up at UOC Neurologia of Fondazione Policlinico Universitario A. Gemelli IRCCS, Rome. All patients were evaluated at the Department of Otolaryngology of Fondazione Policlinico Universitario A. Gemelli IRCCS, Rome, Italy. The study was approved by the local Ethics Committee (approval number ID 6336 CET Lazio Area 3), and all procedures were conducted in accordance with the principles of the Declaration of Helsinki. All participants provided written informed consent for diagnostic testing and the use of anonymized clinical data for research purposes. The study included a cohort of 15 patients (mean age at evaluation: 64.6 years; range: 55–73 years). The mean age of symptom onset was 48.5 years (range: 37–59 years), highlighting a generally late-onset presentation (Table 1). Clinical neurological severity of disease was assessed via the Scale for the Assessment and Rating of Ataxia (SARA) [9]. All included patients were genetically confirmed CANVAS patients as in Cortese et al. [1] and had undergone a complete neuro-otological assessment including clinical examination and video Head Impulse Test (vHIT). Exclusion criteria included the presence of other neurological disorders potentially affecting vestibular function, previous vestibular surgery, active middle or inner ear disease, or incomplete vestibular data. Demographic and clinical information was collected from medical records, including age at evaluation, sex, age at symptom onset, disease duration, presenting symptoms, history of falls, neurological manifestations, oscillopsia, and Scale for the Assessment and Rating of Ataxia (SARA) score [9]. Disease duration was calculated as the interval between motor symptom onset and vestibular evaluation.

**Vestibular Evaluation**: Clinical examination included the assessment of spontaneous and/or positional nystagmus, the head-shaking test (HST), and the bedside head impulse test (HIT). The vHIT was performed as previously described [10]. A lightweight video-oculography (VOG) device (ICS Impulse, GN Otometrics, Taastrup, Denmark) equipped with a high-speed digital camera (250 Hz) was used to assess vestibulo-ocular reflex (VOR) function. The device software (OTOsuite Vestibular Software, Version 120 Build 310) automatically calculated individual and mean VOR gains for each side. Subjects were seated approximately 1.2 m from a fixed visual target. Trained examiners delivered at least 20 unpredictable, small-amplitude (15–20°) horizontal head impulses toward each side, with peak head velocities ranging from 150 to 200°/s. Eye and head velocity traces were recorded and analyzed. VOR gain was automatically calculated by the software as the ratio between the area under the eye velocity curve and the area under the head velocity curve. A mean horizontal canal VOR gain < 0.8 was considered abnormal. In addition to quantitative VOR gain assessment, a qualitative analysis of corrective saccades was performed. Compensatory saccades occurring during head movement were classified as covert, whereas those occurring after completion of the head impulse were classified as overt. Only corrective saccades with peak velocities >100°/s were included in the analysis, as smaller saccades may also be observed in healthy individuals [11,12]. Qualitative analysis focused on the temporal distribution and variability of corrective saccades. Particular attention was paid to the temporal organization of corrective saccades. Traces were classified as showing a clustered (isochronous) pattern when corrective saccades occurred within a relatively narrow latency interval across successive head impulses, and as a dispersed (anisochronous) pattern when corrective saccades exhibited marked latency variability and lacked consistent temporal clustering. For each patient, vHIT recordings from both horizontal semicircular canals were reviewed to assess corrective saccadic behaviour. The analysis included the number of corrective saccades, latency interval (defined as the difference between maximum and minimum saccadic latency), mean saccadic peak velocity, and predominant compensatory pattern (covert or overt). Finally, associations between qualitative saccadic parameters, quantitative VOR gain values, disease duration, and clinical severity assessed using the Scale for the Assessment and Rating of Ataxia (SARA) [9] were explored through correlation analyses.

**Statistical Analysis**: Descriptive statistics were used to summarize demographic, clinical, and vestibular variables. Continuous variables were reported as mean ± standard deviation (SD) or median and range, as appropriate, whereas categorical variables were expressed as absolute numbers and percentages. Disease duration was calculated as the interval between the reported age at motor symptom onset and the age at vestibular evaluation. Correlations between quantitative vestibular parameters (VOR gain), qualitative saccadic parameters (number of corrective saccades, latency interval, and saccadic peak velocity), disease duration, and clinical severity assessed by the Scale for the Assessment and Rating of Ataxia (SARA) [9] were explored using Spearman’s rank correlation coefficient due to the limited sample size and the potential non-normal distribution of variables. Correlation coefficients (r) were interpreted according to conventional thresholds: weak (<0.40), moderate (0.40–0.69), and strong (≥0.70). Statistical significance was set at *p* < 0.05. All statistical analyses were performed using Statistica, Statsoft, Tulsa, OK, USA.

## 3. Results

**Demographics and Clinical Characteristics:** The study included a cohort of 15 patients (mean age at evaluation: 64.6 years; range: 55–73 years). The mean age of symptom onset was 48.5 years (range: 37–59 years), highlighting a generally late-onset presentation. The most common primary presenting symptom was postural instability, reported by most of the cohort, followed by vertigo. A history of falls was confirmed in 12 out of 14 patients who provided this information. Additional neurological symptoms reported in the cohort included muscle pain, paresthesia, tremors, and cough. Signs of distal hypopallesthesia and cerebellar dysarthria were also present in a subset of patients (see Tab 1 for more demographic information). Oscillopsia was reported in 3 of 15 patients (20%). Clinical severity, assessed via the Scale for the Assessment and Rating of Ataxia (SARA) [9], was highly variable among the 12 patients with recorded scores. The SARA scores ranged from 1.5 to 11.5, reflecting a broad spectrum of cerebellar involvement from minimal signs to moderate ataxia. Spontaneous or gaze-evoked nystagmus was observed in 11 patients, with downbeat nystagmus being the predominant pattern (10 patients), while one patient exhibited rotatory nystagmus (Table 1).

**Video Head Impulse Test (vHIT): VOR Gain:** Quantitative evaluation of the high-frequency vestibulo-ocular reflex (VOR) via vHIT demonstrated a wide dispersion of bilateral vestibular function. For the left lateral semicircular canal (n = 15), VOR gain values ranged from normal or hypermetric responses (up to 1.23) to severe deficits indicating profound vestibular areflexia (minimum gain: 0.03). Similarly, the right lateral VOR gain (n = 15) ranged from 0.05 to 1.33. Notably, several patients exhibited severely reduced bilateral VOR gains (ranging from 0.03 to 0.27), consistent with the severe bilateral vestibulopathy characteristic of fully penetrant CANVAS. Finally, the calculated functional asymmetry between the left and right vestibular parameters (n = 15) ranged from 2% to 29%. The generally low-to-moderate asymmetry in most patients (frequently ≤ 20%) further supports the bilateral and relatively symmetric nature of the vestibular impairment in this patient population (Table 2, Figure 1).

**Saccadic Metrics and Asymmetry:** Compensatory oculomotor mechanisms were evaluated through the analysis of catch-up saccades (vHIT). All patients in the cohort demonstrated anisochronous compensatory saccadic recruitment. These were predominantly overt saccades with prolonged latency intervals, with no evidence of covert saccadic patterns or late-latency (>120 ms) isochronous clustering. On the left side, the number of saccades per trial varied significantly (range: 4–26), with amplitudes ranging from 50 to 280 degrees/second (°/s) and latency intervals spanning from 10 to 320 milliseconds (ms). On the right side, the number of saccades ranged from 3 to 36, with amplitudes between 30 and 280 degrees/second and latencies between 50 and 380 milliseconds. Patients with the lowest VOR gains systematically demonstrated the highest saccadic amplitudes (e.g., 280 in patients with gains < 0.30) and longer latency interval distribution, underscoring a robust compensatory saccadic recruitment (Table 2, Figure 1, Figure 2, Figure 3 and Figure 4).

**Impact of Disease Duration on Vestibular Function:** Patients with a longer disease course—calculated from the reported age of onset to the age at the time of evaluation—exhibited a correspondingly higher number of compensatory saccades (Figure 2). The calculated asymmetry between left and right VOR parameters did not significantly correlate with disease duration, further confirming the predominantly symmetric, progressive nature of bilateral vestibular hypofunction in CANVAS.

**Correlations between VOR Gain and Saccadic Metrics:** Correlation analysis revealed a strong interdependence between the severity of the vestibulo-ocular reflex (VOR) deficit and the kinematic parameters of compensatory saccades. Specifically, a marked inverse correlation emerged between VOR gain values and saccadic amplitude (*r* = −0.72, left; *r* = −0.81, right). The progressive deterioration of peripheral vestibular function corresponds to a proportional and robust increase in the magnitude of the corrective eye movement. This relationship highlights how the oculomotor system increases saccadic output in an attempt to compensate for the severe retinal slip exacerbated by vestibular areflexia (Figure 3). Concurrently, a significant negative correlation was observed between VOR gain and the saccadic latency interval (*r* = −0.79, left; *r* = −0.80, right). Patients exhibiting the most severely compromised gain values (e.g., VOR < 0.30) not only generate saccades with larger excursions but also manifest a significantly greater delay in their onset compared to subjects with milder deficits. Furthermore, the data demonstrate a direct positive correlation between the amplitude and latency of the saccades themselves (Figure 4). This intrinsic parametric coupling indicates that larger corrective saccades, deployed in response to a collapsed vestibular signal, are structurally associated with more prolonged recruitment intervals. Taken together, these results objectively capture the specific oculomotor signature of the examined patients: a compensatory saccadic recruitment with an anisochronous morphology, where the degree of vestibular deafferentation strictly dictates both the temporal delay and the energy of the compensatory impulse. This architecture consolidates the evidence of an isolated and delayed overt pattern, characterized by wide latency intervals and the complete absence of early saccadic clusters or isochronous groupings.

**Clinical-Instrumental Correlations (SARA Score and vHIT):** To assess the relationship between clinical severity and instrumental findings, we evaluated correlations between the Scale for the Assessment and Rating of Ataxia (SARA) score and vHIT parameters. While bilateral vestibular areflexia is a hallmark of CANVAS, the magnitude of VOR gain reduction showed a variable degree of correlation with the SARA score, suggesting that vestibular deterioration and cerebellar ataxia may progress at independent rates in a subset of patients. However, the presence of specific oculomotor abnormalities, such as downbeat nystagmus, was frequently observed in patients exhibiting higher SARA scores alongside severe bilateral vestibulopathy. Overall, our findings underscore the complex pathophysiological interplay between cerebellar and vestibular degeneration in CANVAS. The robust negative correlation between VOR gain and saccadic amplitude highlights the active, though often insufficient, compensatory mechanisms deployed by the oculomotor system. Notably, the dissociation between the progression of the SARA score and the decline in VOR gain in some patients supports the hypothesis that the sensory neuronopathy, cerebellar ataxia, and vestibular hypofunction in CANVAS may follow distinct longitudinal trajectories. These results emphasize the necessity of combining quantitative vHIT assessments with clinical scoring to monitor disease progression.

## 4. Discussion

The present study provides a combined quantitative and qualitative characterization of vHIT findings in CANVAS patients. The main findings are: (1) severe bilateral vestibular hypofunction, (2) progressive reduction in VOR gain with increasing disease duration, (3) strong associations between vestibular impairment and corrective saccadic metrics, and (4) a predominantly overt and temporally dispersed compensatory saccadic pattern.

Our results confirm that vestibular impairment in CANVAS is severe, bilateral, and largely symmetric. Most patients exhibited markedly reduced VOR gains—often approaching complete areflexia—with low interaural asymmetry. Because this asymmetry did not correlate with disease duration, CANVAS appears characterized by a diffuse, symmetric degeneration of vestibular afferents rather than a fluctuating or unilateral process. This supports its classification as a bilateral vestibular ganglionopathy within the RFC1-related spectrum [2,13], aligning with previous reports of abnormal vHIT in 73–90% of patients [1,3,4,6,14].

We also observed a significant inverse relationship between disease duration and VOR gain, paralleled by increased corrective saccade amplitude and frequency. While our cross-sectional design precludes defining these parameters as definitive progression biomarkers, they suggest that vHIT reflects the cumulative burden of vestibular deafferentation over time [2]. Furthermore, this high-frequency vHIT deficit complements literature demonstrating severe low-frequency impairment on caloric testing [6] and impaired visual–vestibular integration on VVOR [7,8]. Together, these findings indicate a global, non-frequency-specific failure of vestibular afferent signaling. This widespread vestibular failure provides the physiological substrate for the abnormal saccadic organization observed in our cohort.

A novel finding is the robust correlation between VOR gain reduction and saccadic kinematics. Lower VOR gains were associated with larger corrective saccades and increased latency, while amplitude and latency were themselves positively correlated. Physiologically, severe vestibular hypofunction generates increasing retinal slip, demanding larger and more frequent compensatory eye movements to restore gaze stability. The concomitant increase in latency indicates that compensatory mechanisms become not only amplified but also temporally delayed in advanced deafferentation. This temporal–kinematic coupling highlights the clinical value of qualitative vHIT analysis beyond conventional VOR gain measurements [10].

Crucially, all patients exhibited a predominantly overt, temporally dispersed (anisochronous) saccadic pattern, with marked latency variability and absent covert saccade clustering. This reflects the dual pathology of CANVAS: peripheral vestibular loss combined with cerebellar degeneration. Because the cerebellum mediates saccadic timing and adaptation, its impairment disrupts central oculomotor calibration. The combination of vestibular failure, defective VVOR, and cerebellar timing dysfunction forces a compensatory strategy relying on overt, delayed, and dispersed corrective saccades. We hypothesize this distinct oculomotor behavior represents a characteristic functional signature of CANVAS, emerging from the interaction between neuronopathy and cerebellar dysfunction.

Although vestibular impairment was universally severe, its correlation with clinical severity (SARA score) was not strictly linear. This dissociation suggests that cerebellar ataxia and vestibular hypofunction progress at partially independent rates [15,16]. Inter-individual variability may also reflect differing repeat expansion sizes across the RFC1 spectrum [17]. Nonetheless, patients with advanced vestibular impairment frequently showed additional oculomotor deficits, such as downbeat nystagmus, reaffirming that vestibular and cerebellar degenerations interact while maintaining distinct pathological trajectories.

Interestingly, despite the marked horizontal VOR impairment observed in our cohort, oscillopsia was reported by only three patients (20%). This apparent discrepancy may reflect individual differences in visual dependence, central adaptation, symptom perception, and functional compensation. However, the small number of patients reporting oscillopsia and the lack of a standardized symptom scale preclude any firm conclusions regarding the relationship between subjective visual instability and objective vestibular impairment. Future prospective studies using standardized assessment of oscillopsia and visual function may help clarify this relationship.

This study presents several limitations. First, the retrospective, monocentric design and small sample size limit the generalizability of our findings, while the lack of longitudinal follow-up precludes conclusions regarding individual disease trajectories. Furthermore, the qualitative evaluation of corrective saccades was not based on a formally validated scoring system.

Second, our analysis focused exclusively on horizontal vHIT responses. Although this approach facilitated a standardized quantitative and qualitative assessment, the absence of vertical canal evaluation remains a limitation. While vertical canal measurements can be technically challenging and exhibit greater variability [18,19], their inclusion is necessary to determine whether the qualitative saccadic pattern observed in our cohort also occurs during vertical canal stimulation.

Third, complementary vestibular tests were not systematically performed. Caloric testing was available only for a limited subset, preventing meaningful comparison with vHIT data, and rotatory chair testing was unavailable, although not required by current diagnostic criteria. Similarly, the absence of vestibular evoked myogenic potentials (VEMPs) and posturography prevents us from drawing independent conclusions regarding otolithic or postural function, despite prior studies suggesting relatively preserved otolithic function in CANVAS [20].

Future prospective studies with larger cohorts, incorporating systematic assessments of all six semicircular canals alongside caloric, rotational, and otolithic testing, are essential to fully characterize the extent and frequency-specific nature of vestibular dysfunction in RFC1-related disorders.

## 5. Conclusions

In conclusion, CANVAS features severe, symmetric bilateral vestibular hypofunction with a distinctive oculomotor profile. Beyond isolated VOR gain reduction, qualitative vHIT analysis reveals overt, temporally dispersed corrective saccades whose kinematics strongly depend on vestibular loss severity. This qualitative assessment provides crucial, complementary information for the characterization of vestibular dysfunction in CANVAS.

## Figures and Tables

**Figure 1:**
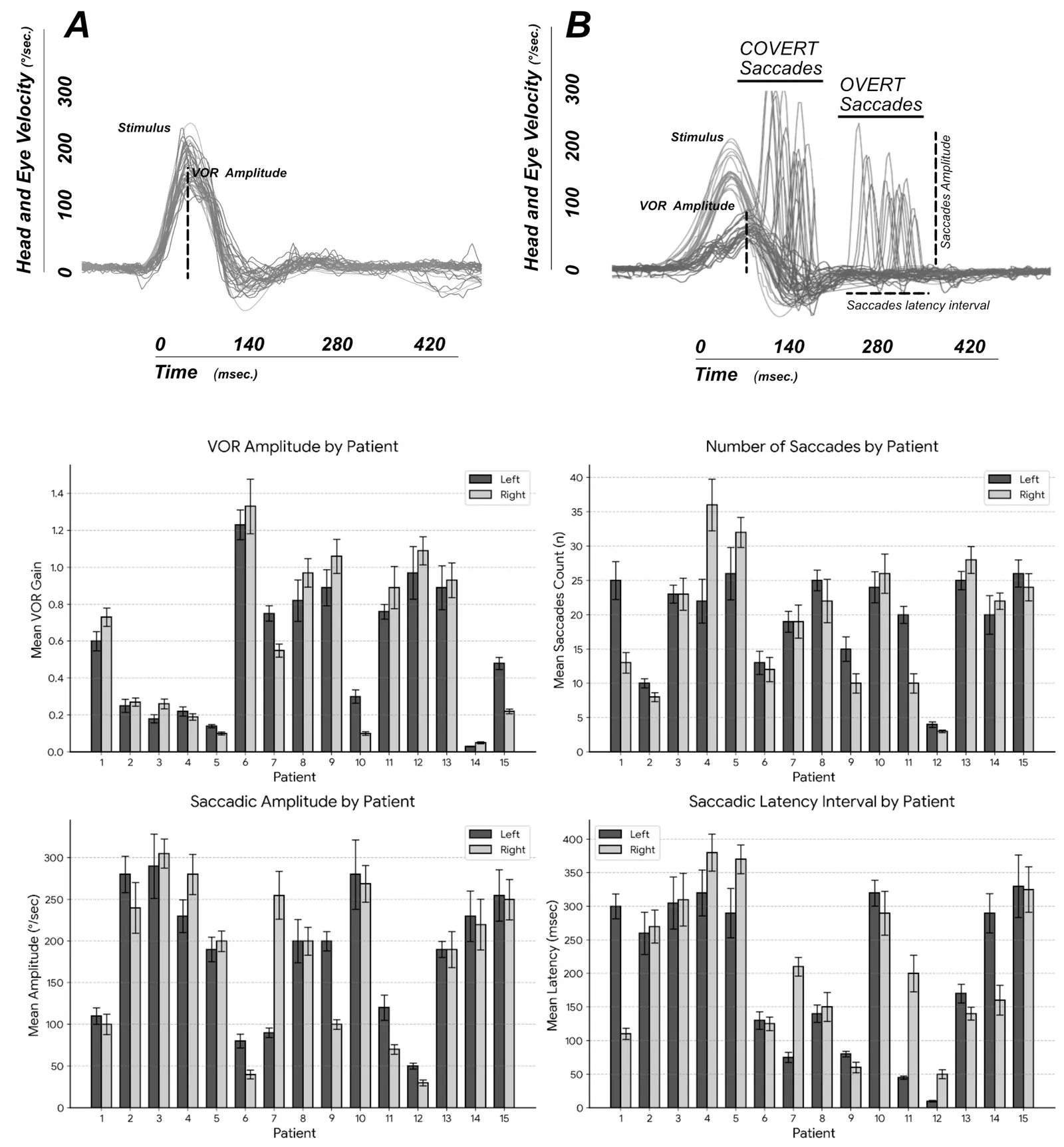
Qualitative and quantitative characterization of video head impulse test (vHIT) responses in CANVAS patients. (**A**) Normal vHIT response. (**B**) Example of pathological vHIT trace: dotted lines indicate the specific kinematic and temporal parameters extracted for qualitative analysis. Grouped bar charts displaying the detailed vHIT metrics for the entire patient cohort (n = 15), analyzed for the left (dark gray bars) and right (light gray bars) sides. The panels illustrate the marked inter-individual variability regarding Mean VOR Gain (top left), Mean Saccades Count (top right), Mean Saccadic Amplitude (bottom left), and Mean Saccadic Latency Interval (bottom right).

**Figure 2:**
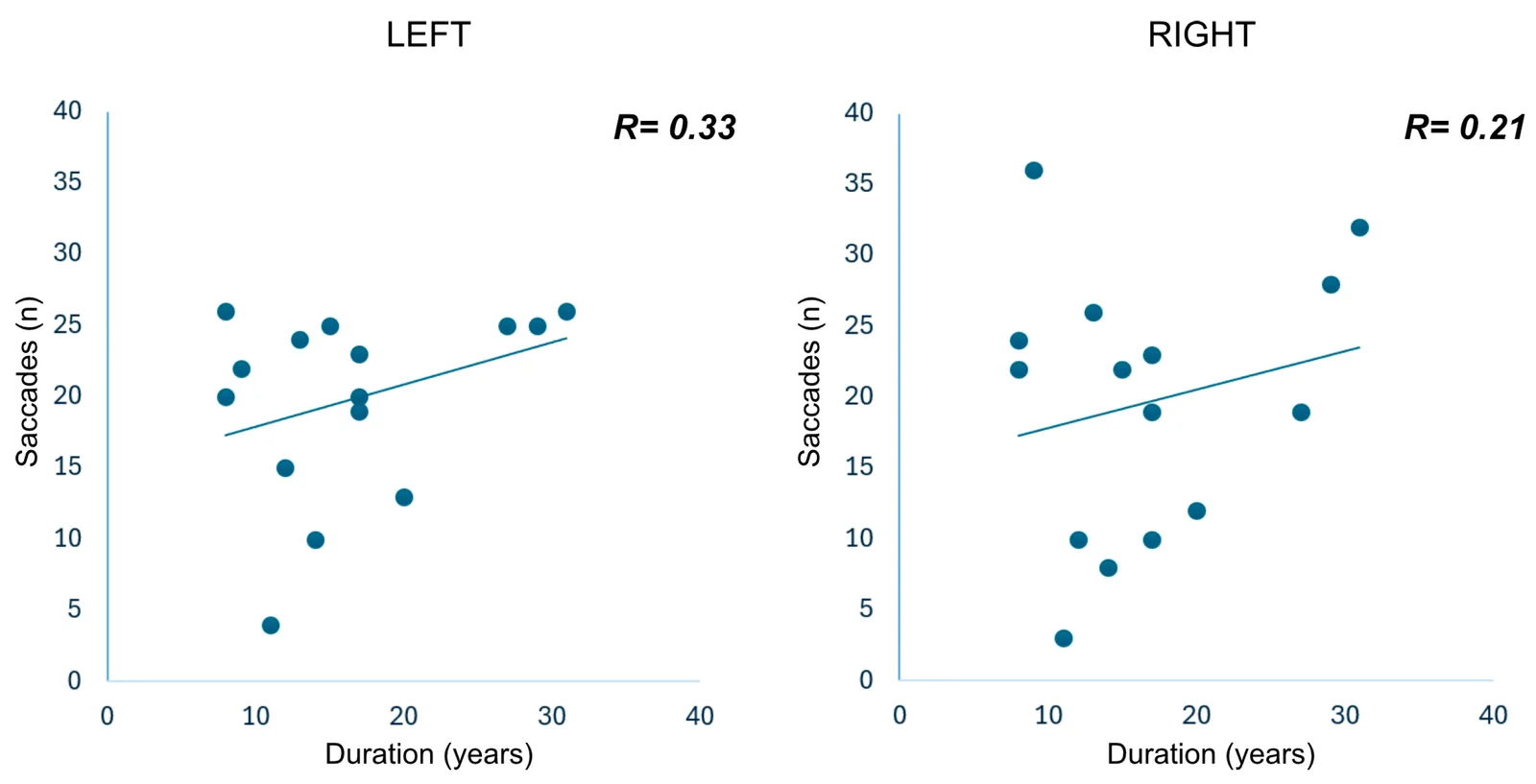
Relationship between disease duration and corrective saccadic recruitment. Scatterplots evaluating the correlation between disease duration (in years) and the mean number of compensatory saccades generated during vHIT testing for the left (R = 0.33) and right (R = 0.21) sides. The slight positive trend lines suggest a progressive increase in saccadic recruitment over time as the cumulative burden of vestibular deafferentation advances.

**Figure 3:**
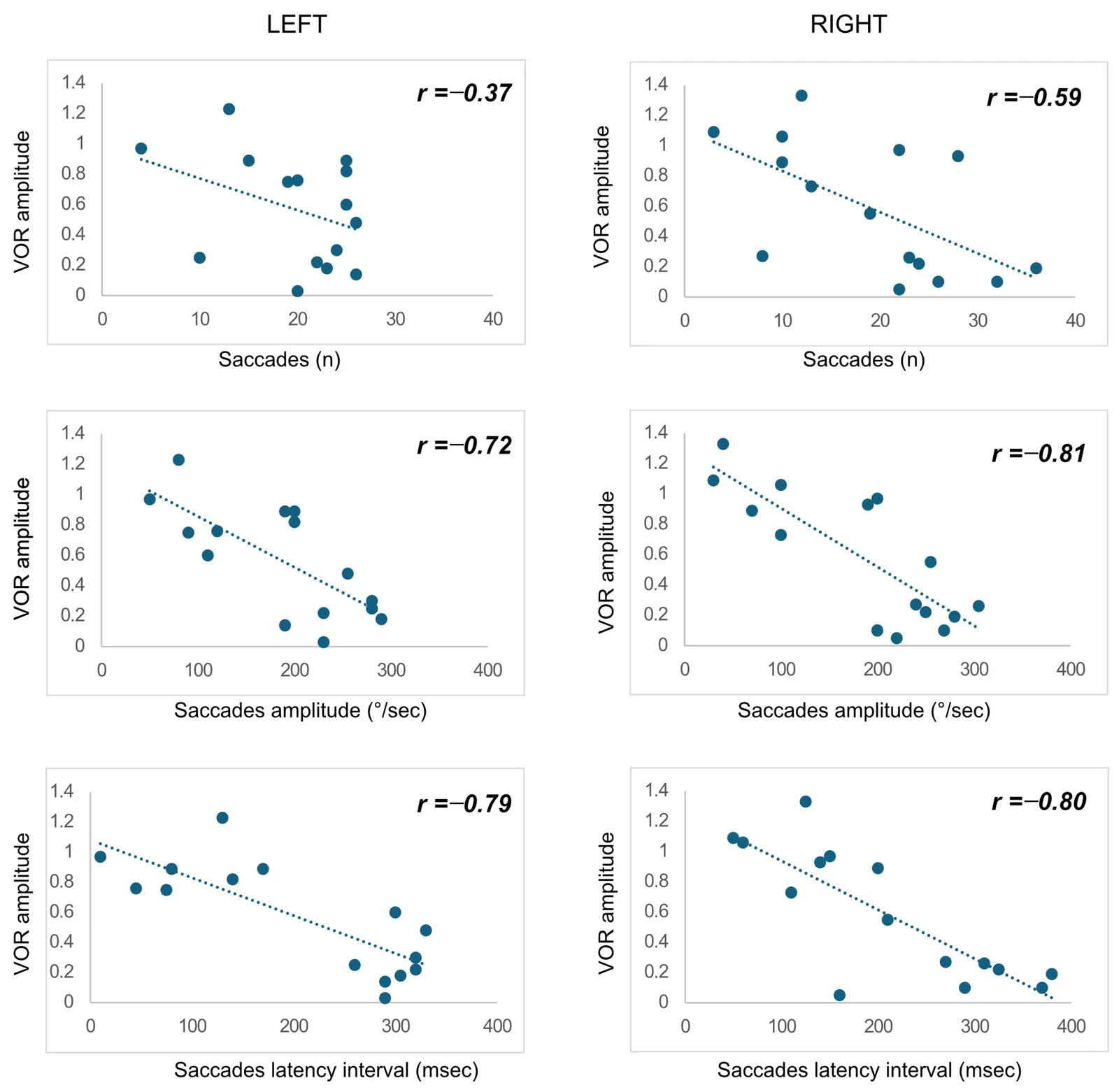
Correlation between Vestibulo-Ocular Reflex (VOR) amplitude and compensatory saccadic metrics. Scatterplots illustrating the inverse relationship between VOR amplitude and the number of saccades (**top** row), saccadic amplitude (**middle** row), and saccadic latency interval (**bottom** row) for both the left and right sides. The strong negative correlations demonstrate that severe vestibular hypofunction drives a robust compensatory response; specifically, maximum saccadic amplitudes (up to 280°/s) and prolonged latency intervals systematically correspond to the lowest VOR values.

**Figure 4:**
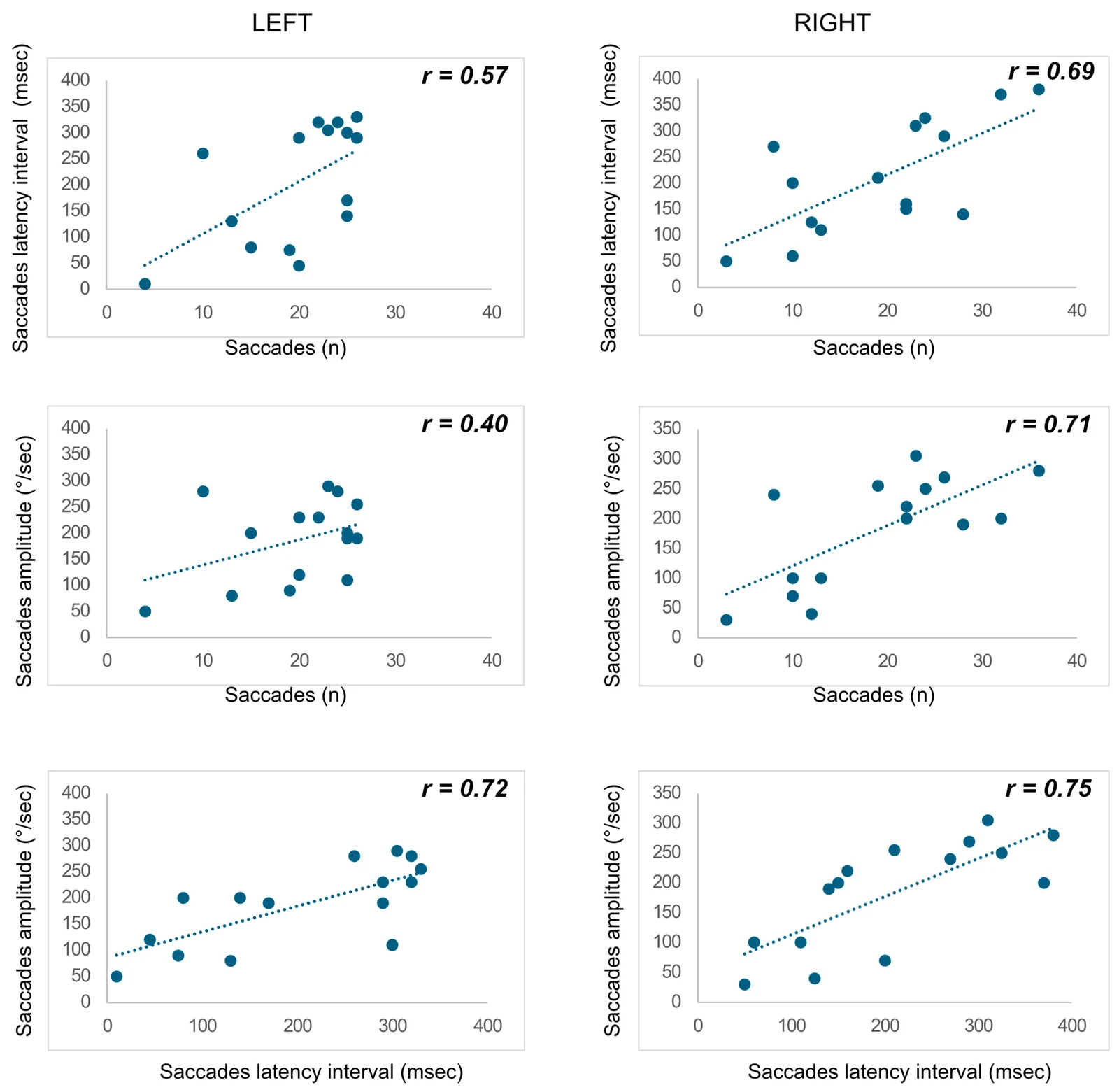
Kinematic and temporal associations characterizing the compensatory saccadic pattern. Scatterplots demonstrating the internal correlations among qualitative saccadic parameters for the left and right sides. The panels display the relationships between saccadic latency interval and saccade count (**top** row), saccadic amplitude and saccade count (**middle** row), and saccadic amplitude versus latency interval (**bottom** row). The consistent positive correlations highlight an anisochronous compensatory recruitment strategy, where an increased number of predominantly overt saccades is coupled with both larger kinematic amplitudes and temporally dispersed, delayed-onset latencies.

**Table 1:** Demographic and Clinical Characteristics of the Patient Cohort.

Total patients (n)	15
Age at evaluation (years), mean (range)	64.6 (55–73)
Age of symptom onset (years), mean (range)	48.5 (37–59)
Clinical Presentation & History	
Most common primary symptoms	Postural instability, Vertigo
History of falls (n/total evaluated)	12/14
Additional reported symptoms	Muscle pain, paresthesia, tremors, cough
Neurological signs	Distal hypopallesthesia, cerebellar dysarthria
Documented Oscillopsia	3/14
Clinical Severity & Examination	
SARA score, range (n = 12)	1.5–11.5
Presence of nystagmus (n)	11
Downbeat nystagmus	*10*
Rotatory nystagmus	*1*

**Table 2:** Quantitative and qualitative video head impulse test (vHIT) findings.

Parameter	Side (n = 15)	Range (Min–Max)	Key Observations & Saccadic Morphology
**VOR Gain**	Left Right	0.03–1.23 0.05–1.33	Wide functional dispersion. Fully penetrant CANVAS cases exhibit severe bilateral vestibulopathy (gain range: 0.03–0.27).
**Functional Asymmetry**		2–29%	Generally low-to-moderate (frequently ≤ 20%), confirming a relatively symmetric bilateral impairment.
**Saccades Count (n)**	Left Right	4–26 3–36	Anisochronous compensatory recruitment. Predominantly overt saccades. No evidence of covert patterns or late latency (>120 ms) isochronous clustering.
**Saccadic Amplitude** (°/s)	Left Right	50–280 30–280	Robust compensatory drive: maximum amplitudes (up to 280°/s) systematically correspond to the lowest VOR gains (<0.30).
**Saccadic Latency** (ms)	Left Right	10–320 50–380	Prolonged latency interval distribution, typically observed in patients with severely reduced VOR gains.

## Data Availability

The data presented in this study are available on request from the corresponding author, in compliance with the authors’ institute’s rules on the processing of scientific and personal data.
